# Association between remnant cholesterol and heart failure: A prospective cohort study

**DOI:** 10.3389/fcvm.2022.938647

**Published:** 2022-10-28

**Authors:** Heng Liu, Jing Zhang, Zhangbin Li, Jie Liu, Shuping Lian, Jianhua Le

**Affiliations:** Department of Cardiology, Heyuan People’s Hospital, Heyuan, China

**Keywords:** remnant cholesterol, heart failure, (ARIC) study, general population, prospective cohort study

## Abstract

**Background:**

Elevated remnant cholesterol (RC) is associated with a higher risk of various cardiac diseases. Heart failure (HF) usually occurs at the end stage of various cardiac diseases. However, there is limited research on the association between RC and the risk of HF. Therefore, we aimed to provide relevant evidence by determining whether a high RC level also influences the risk of HF.

**Materials and methods:**

In this secondary analysis of the Atherosclerosis Risk in Communities (ARIC) study, we included 12,595 participants without coronary heart disease. We determined the association of the RC level as a continuous or categorical variable with the risk of HF using the multivariable-adjusted Cox proportional hazards models and restricted cubic spline curve.

**Results:**

During a median follow-up of 22.5 years, 2,029 (16.1%) cases of HF occurred in all included participants. Compared with participants in the RC < 0.50 mmol/L group, the adjusted hazard ratio (HR) for HF increased progressively in participants with the RC level of 0.50 to 0.99 mmol/L, 1.00 to 1.49 mmol/L, and ≥1.50 mmol/L, from 1.17 (95% confidence interval [CI]: 1.05–1.30) to 1.27(95% CI: 1.08–1.49) and to 1.50 (95% CI: 1.14–1.97) (*P* for trend < 0.001). Cubic spline curves also revealed that the risk of HF increased with the RC level.

**Conclusion:**

In the general population without coronary heart disease, a higher level of RC was significantly associated with a higher risk of HF, indicating that a higher RC level might be a potential risk factor for HF. Therefore, the management of blood cholesterol to reduce the risks of HF should focus not only on the traditional blood lipid parameters but also on the RC level.

## Introduction

The worldwide prevalence of heart failure (HF) is increasing as the treatment of myocardial infarction and ischemic heart disease has improved and as diabetes mellitus and obesity have increased in prevalence (1, 2). HF is a complicated syndrome that usually occurs at the end stage of various cardiac diseases and has high morbidity and mortality with no curable treatment for most patients at present time (1, 2). Therefore, it is especially critical to reduce the risk of HF by identifying the potential risk factors and managing them.

Previous studies have shown that decreased high-density lipoprotein cholesterol (HDL-c) and elevated non-HDL-cholesterol were associated with an increased risk of future HF (3, 4). Furthermore, in the primary- and secondary-prevention trials, statins therapy reduced low-density lipoprotein cholesterol (LDL-c) and the risks of HF in patients with or without myocardial infarction (5). However, there are still a great number of patients who have received statins therapy, suffering from HF events (5). Thus, the traditional blood lipid parameters might fail to represent the full spectrum of lipid-related HF risk. Recently, a growing body of research focused on the adverse effects of remnant cholesterol (RC) (6). RC was cholesterol-rich in triglyceride lipoprotein, which consists of chylomicron remnants in the non-fasting state, and intermediate-density lipoproteins and very-low-density lipoproteins in the fasting state (7). Several studies have shown that the elevated level of RC in serum was associated with the increased risks of cardiovascular disease, aortic valve stenosis, and diabetes (8–11). However, the effects of RC on subsequent HF events remain unknown, which impedes the development of an optimum approach for the management of blood lipid.

Therefore, our study aimed to identify the association of RC with the risks of HF using data from the Atherosclerosis Risk in Communities (ARIC) study (12).

## Materials and methods

### Study design and study population

The ARIC study is a prospective cohort study designed to assess the risk factors for cardiovascular disease. A total of 15,792 participants aged 45–64 were recruited from four US population centers between 1987 and 1989: Forsyth County, North Carolina; Jackson, Mississippi; Washington County, Maryland; and Northwestern suburbs of Minneapolis, Minnesota. Participants were examined initially every 3 years for the three subsequent visits, with the second examination in 1990–1992 (visit 2), the third in 1993–1995 (visit 3), and the fourth in 1996–1998 (visit 4). The fifth visit took place between 2011 and 2013 after fifteen years. Details of the study design have been published elsewhere (12).

In this study, 12,595 participants were included in the final analyses. We excluded those missing data in the public access data sets (*n* = 809), those missing information on covariates (*n* = 706), those with coronary heart disease at baseline (*n* = 547), and those missing information on HF events (*n* = 1,135) (Supplementary Figure 1). The ARIC study program was approved by institutional review boards at all four centers, and informed consent was obtained from all participants.

### Measurement of remnant cholesterol

Observational analyses were conducted with RC as exposure. RC was calculated as total cholesterol minus LDL-c minus HDL-c, as done previously (13).

### Covariate measurement

Recorders obtained information on characteristics that could influence our outcome, including age, sex, race and education level (self-reported), smoking status (Current indicates a participant report of current cigarette use; Former indicates any report of cigarette use at a previous study visit or of past cigarette use; Never), drinking status (Current indicates any alcohol consumption within the last 6 months; Former indicates any previous alcohol consumption if none was consumed within the last 6 months; Never). Height (m) and weight (kg) were measured with the participant wearing light clothes by trained staff, and body mass index (BMI) was calculated as weight divided by squared height (kg/m^2^). Total cholesterol, triglycerides, and HDL-c were measured with enzymatic assays. The estimated glomerular filtration rate (eGFR) was based on the creatinine-based Chronic Kidney Disease Epidemiology Collaboration Equation (14). Systolic blood pressure (SBP), diastolic blood pressure (DBP), and fasting glucose were also recorded. Hypertension was defined as SBP ≥ 140 mmHg and/or DBP ≥ 90 mmHg, or blood pressure medication use in the past 2 weeks. Diabetes mellitus was defined as fasting blood glucose ≥ 126 mg/dL or non-fasting blood glucose ≥ 200 mg/dL, use of antidiabetic medicines, or self-reported physician diagnosis of diabetes mellitus. Medication histories (aspirin, cholesterol-lowering medication, antihypertensive medication) were adjudicated when the participants were reported to have taken these medications within the last 2 weeks or taking these medications.

### Heart failure ascertainment

The primary outcome of interest was incident HF, defined as an HF-associated hospitalization or HF-associated death occurring after the baseline examination (visit 1). Prior to 2005, the ARIC study did not collect record material other than discharge codes for incident HF hospitalizations. Thus, it was defined by diagnostic code [International Classification of Diseases, Ninth Revision (ICD-9) code 428] from hospital discharges until 2004 (15). After 2005, ARIC staff members abstracted a broad range of hospital records for potential HF events to ascertain HF hospitalization (16).

### Statistical analyses

Baseline characteristics are presented as the mean (standard deviation [SD]) for continuous variables or number (%) for categorical variables. All included participants were classified into four groups according to the RC level at baseline (<0.50 mmol/L, 0.50–0.99 mmol/L, 1.00–1.49 mmol/L, and ≥1.50 mmol/L). We constructed Kaplan–Meier graphs and used the log-rank test to assess differences in the cumulative incidence of HF among four groups. The multivariable-adjusted Cox proportional hazards models were used to estimate the hazard ratio (HR) (95% confidence intervals [CIs]) for HF associated with the RC level, including the following covariates: model 1: age, sex, race; model 2: variables in model 1 plus education level, BMI, smoking status, drinking status, SBP, DBP, eGFR, prevalent hypertension, diabetes mellitus, and medication histories of aspirin and antihypertensive drugs; and model 3: variables in model 2 plus total cholesterol, LDL-C, cholesterol-lowering medication. The continuous association between RC and the risks of HF was also assessed by using multivariable-adjusted Cox proportional hazards models and the restricted cubic spline models with three knots.

Subgroup analyses of key variables (age, sex, race, total cholesterol, LDL-c, hypertension, diabetes, and smoking status) were also used to assess the effect stratified by prespecified risk factors and the potential interaction effect. An interaction term between key variable and RC was individually added to the adjusted Cox model 3, and the *P*-values and HR (CIs) for these associations were estimated. To avoid the impact of cholesterol-lowering medication on the association of RC with the risk of HF, we also conducted a sensitivity analysis in participants without using cholesterol-lowering medication (*n* = 12,290). All the tests were two-sided with *P* < 0.05 considered significant. Statistical analyses were conducted using the SPSS 20.0 (IBM Inc., Armonk, NY, USA) and the Stata Version 14 (StataCorp, College Station, TX, USA).

## Results

### Baseline characteristics of participants

The baseline characteristics of participants are shown in Table 1. Among the 12,595 participants with an average of 54.4 years from the ARIC study, 44.7% were male, and 24.1% were black. The long-term changes of serum cholesterol (total cholesterol, LDL-c, HDL-c, and RC) from visit 1 to 5 are shown in Supplementary Figure 2. Serum cholesterol levels are nearly constant during visit 1 to 5, especially the RC level. Compared with lower RC level groups (<0.50 mmol/L and 0.50–0.99 mmol/L), participants with RC ≥ 1.5 mmol/L were more likely to be men and White, to have a higher BMI level, a higher SBP and DBP level, a higher total cholesterol level, a higher triglyceride level, a higher fasting glucose level, and comorbidity (hypertension, diabetes), and to use aspirin, antihypertensive medication, and cholesterol-lowering medication.

**TABLE 1:** Baseline characteristics of each group categorized by remnant cholesterol (mmol/L).

Characteristics	Total	<0.50	0.50–0.99	1.00–1.49	≥1.50	*P*-value
No.	12595	5259	5701	1286	349	
Age, years	54.4 (5.7)	53.4 (5.8)	54.5 (5.7)	54.9 (5.6)	54.4 (5.4)	<0.001
**Sex, No. (%)**						<0.001
Men	5635 (44.7)	2082 (39.6)	2656 (46.6)	704 (54.7)	193 (55.3)	
Women	6960 (55.3)	3177 (60.4)	3045 (53.4)	582 (45.3)	156 (44.7)	
**Race, No. (%)**						<0.001
Black	3034 (24.1)	1546 (29.4)	1259 (22.1)	185 (14.4)	44 (12.6)	
White	9561 (75.9)	3713 (70.6)	4442 (77.9)	1101 (85.6)	305 (87.4)	
BMI, kg/m^2^	27.4 (5.2)	26.2 (5.0)	28.1 (5.3)	29.0 (4.8)	29.5 (4.6)	<0.001
SBP, mm Hg	120.8 (18.6)	118.7 (19.2)	121.5 (18.1)	124.6 (17.9)	125.4 (18.1)	<0.001
DBP, mm Hg	73.5 (11.1)	72.8 (11.4)	73.7 (10.9)	75.0 (10.8)	75.4 (10.6)	<0.001
Total cholesterol, mmol/L	5.5 (1.1)	5.2 (1.0)	5.7 (1.0)	6.0 (1.1)	6.3 (1.3)	<0.001
HDL-c, mmol/L	1.4 (0.4)	1.6 (0.4)	1.3 (0.4)	1.1 (0.3)	1.0 (0.3)	<0.001
LDL-c, mmol/L	3.5 (1.0)	3.3 (0.9)	3.8 (1.0)	3.8 (1.0)	3.6 (1.2)	<0.001
Triglyceride, mmol/L	1.4 (0.7)	0.8 (0.2)	1.5 (0.3)	2.6 (0.3)	3.8 (0.3)	<0.001
Remnant cholesterol, mmol/L	0.6 (0.3)	0.4 (0.1)	0.7 (0.1)	1.2 (0.1)	1.7 (0.2)	<0.001
eGFR, mL/min/1.73 m^2^	102.5 (15.3)	104.7 (14.9)	101.3 (15.4)	99.4 (15.6)	100.5 (14.8)	<0.001
Fasting glucose, mmol/L	5.9 (2.0)	5.6 (1.5)	6.0 (2.1)	6.6 (2.9)	7.2 (3.5)	<0.001
Diabetes mellitus, No. (%)	1282 (10.2)	318 (6.0)	598 (10.5)	263 (20.5)	103 (29.5)	<0.001
Hypertension, No. (%)	3951 (31.4)	1326 (25.2)	1900 (33.3)	557 (43.3)	168 (48.1)	<0.001
**Education level, No. (%)**						<0.001
Basic or 0 y	2756 (21.9)	1099 (20.9)	1282 (22.5)	307 (23.9)	68 (19.5)	
Intermediate	5222 (41.5)	2050 (39.0)	2444 (42.9)	566 (44.0)	162 (46.4)	
Advanced	4617 (36.7)	2110 (40.1)	1975 (34.6)	413 (32.1)	119 (34.1)	
**Smoking, No. (%)**						<0.001
Current smoker	3286 (26.1)	1222 (23.2)	1617 (28.4)	369 (28.7)	78 (22.3)	
Former smoker	4010 (31.8)	1592 (30.3)	1818 (31.9)	458 (35.6)	142 (40.7)	
Never smoker	5299 (42.1)	2445 (46.5)	2266 (39.7)	459 (35.7)	129 (37.0)	
**Drinking, No. (%)**						0.001
Current drinker	7291 (57.9)	3099 (58.9)	3210 (56.3)	771 (60.0)	211 (60.5)	
Former drinker	2248 (17.8)	888 (16.9)	1051 (18.4)	234 (18.2)	75 (21.5)	
Never drinker	3056 (24.3)	1272 (24.2)	1440 (25.3)	281 (21.9)	63 (18.1)	
Aspirin, No. (%)	5743 (45.6)	2242 (42.6)	2656 (46.6)	644 (50.1)	201 (57.6)	<0.001
Antihypertensive, no. (%)	3189 (25.3)	1027 (19.5)	1551 (27.2)	473 (36.8)	138 (39.5)	<0.001
Cholesterol-lowering medication, No. (%)	305 (2.4)	77 (1.5)	147 (2.6)	54 (4.2)	27 (7.7)	<0.001

Continuous variables are presented as mean (SD), and categorical variables are presented as percentages. BMI: body mass index; SBP: systolic blood pressure; DBP: diastolic blood pressure; HDL-c: high-density lipoprotein cholesterol; LDL-c: low-density lipoprotein cholesterol; eGFR: estimated glomerular filtration rate.

### Association of remnant cholesterol with the risk of heart failure

**FIGURE 1:**
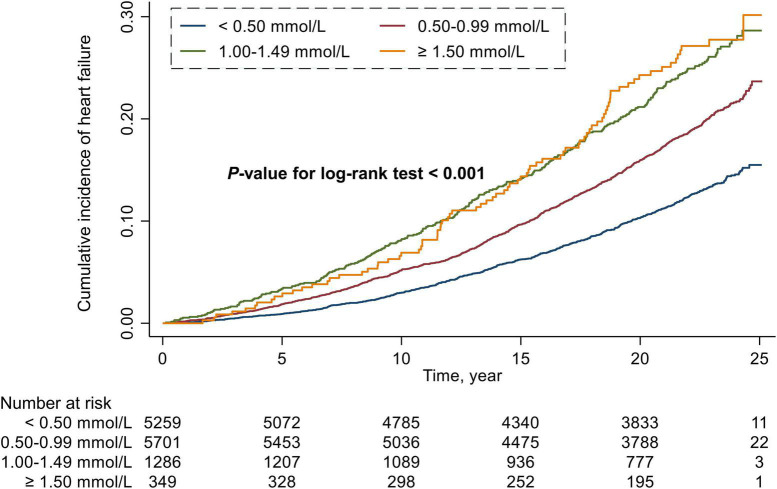
Cumulative incidence estimates (Kaplan–Meier) for heart failure in four groups categorized by the level of remnant cholesterol.

During a median follow-up period of 22.5 years, 2,029 (16.1%) cases of HF occurred in all included participants, including 633 (12.0%) in the RC < 0.50 mmol/L group, 1,019 (17.9%) in the RC of 0.50 to 0.99 mmol/L group, 295 (22.9%) in the RC of 1.00 to 1.49 mmol/L group, and 82 (23.5%) in the RC ≥ 1.50 mmol/L group. The Kaplan-Meier survival function curves showed higher risk of incident HF in individuals with the RC of 0.50 to 0.99 mmol/L, 1.00 to 1.49 mmol/L, and ≥1.50 mmol/L compared with individuals with the RC < 0.50 mmol/L (*P*-value for log-rank test < 0.001) (Figure 1).

**FIGURE 2:**
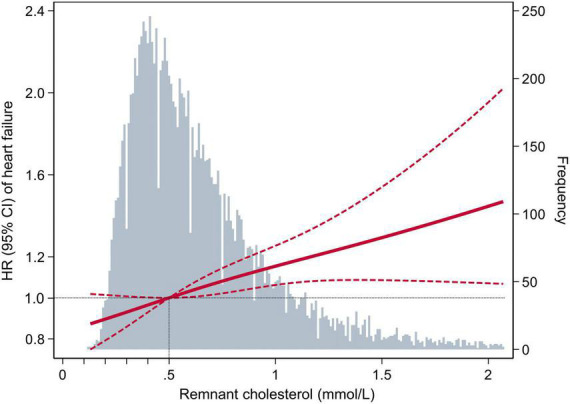
Adjusted hazard ratios (95% confidence interval) for the association of the remnant cholesterol level with the risk of heart failure. Hazard ratios (indicated by a dark-red solid line) and 95% confidence intervals (dark-red dotted lines) are derived from the multivariable-adjusted Cox regression model, adjusted for age, sex, race, education level, body mass index, smoking status, drinking status, systolic blood pressure, diastolic blood pressure, estimated glomerular filtration rate, prevalent hypertension, prevalent diabetes mellitus, use of aspirin and antihypertensive drugs, total cholesterol, low-density lipoprotein cholesterol, use of cholesterol-lowering medication. The remnant cholesterol level was centered at 0.5 mmol/L and modeled using a restricted cubic spline with knots at the 5th, 50th, and 95th percentiles. The black dotted line is the reference line as hazard ratio = 1. Histograms represent the frequency distribution of remnant cholesterol.

In the multivariable-adjusted Cox model, compared with the participants with RC < 0.50 mmol/L, the HR (CIs) for incident HF in the participants with RC of 0.50 to 0.99 mmol/L, 1.00 to 1.49 mmol/L, and ≥1.50 mmol/L were 1.16 (95% CI: 1.05–1.29), 1.26 (95% CI: 1.08–1.48), and 1.49 (95% CI: 1.14–1.94), respectively (*P* for trend < 0.001) (Table 2). We further assessed the continuous association between RC and the risks of HF, and found that an increase of 0.33 mmol/L in RC (corresponding to 1-SD) was also associated with a 10% higher risk of HF (HR, 1.10; 95% CI, 1.04–1.15) (Supplementary Table 1). Cubic spline curves between the RC level and the HR of incident HF are presented in Figure 2 and reveal that the risk of HF increased with the RC level.

**TABLE 2:** Association of remnant cholesterol with the risk of heart failure.

Remnant cholesterol (mmol/L)	Model 1		Model 2		Model 3	
			
	Hazard ratio (95% CI)	*P*-value	Hazard ratio (95% CI)	*P*-value	Hazard ratio (95% CI)	*P*-value
<0.50	1 (ref.)	—	1 (ref.)	—	1 (ref.)	—
0.50–0.99	1.51 (1.36–1.67)	<0.001	1.16 (1.05–1.29)	0.004	1.16 (1.05–1.29)	0.005
1.00–1.49	2.12 (1.84–2.44)	<0.001	1.22 (1.05–1.41)	0.008	1.26 (1.08–1.48)	0.004
≥1.50	2.38 (1.89–3.00)	<0.001	1.38 (1.09–1.75)	0.008	1.49 (1.14–1.94)	0.003
*P* for trend	<0.001		<0.001		<0.001	

Model 1: adjusted for age, sex, race;

Model 2: adjusted for model 1 + education level, body mass index, smoking status, drinking status, systolic blood pressure, diastolic blood pressure, estimated glomerular filtration rate, prevalent hypertension, prevalent diabetes mellitus, use of aspirin and antihypertensive drugs.

Model 3: adjusted for model 2 + total cholesterol, low-density lipoprotein cholesterol, use of cholesterol-lowering medication.

**FIGURE 3:**
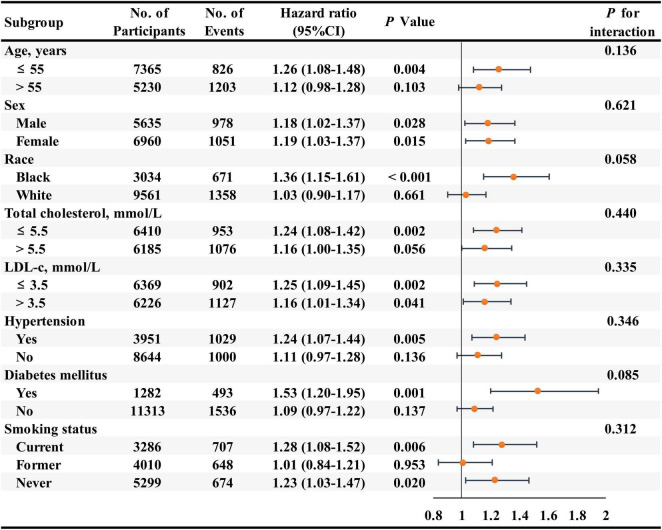
Association of the remnant cholesterol level ≥ 0.50 mmol/L group compared with the remnant cholesterol level < 0.50 mmol/L group for the risks of heart failure in key subgroups. Hazard ratios (95% confidence intervals) were obtained after individually removing the original variable from the multivariable-adjusted Cox model, adjusted for age, sex, race, education level, body mass index, smoking status, drinking status, systolic blood pressure, diastolic blood pressure, estimated glomerular filtration rate, prevalent hypertension, prevalent diabetes mellitus, use of aspirin and antihypertensive drugs, total cholesterol, low-density lipoprotein cholesterol, use of cholesterol-lowering medication.

In addition, we combined the participants with RC of 0.50 to 0.99 mmol/L, 1.00 to 1.49 mmol/L, and ≥1.50 mmol/L into one group to conduct the subgroup analyses due to the higher HF risk for them compared with the participants with RC < 0.50 mmol/L. In subgroup analyses of key variables (age, sex, race, total cholesterol, LDL-c, hypertension, diabetes, and smoking status), although the different risks of HF in the subgroups of age, race, total cholesterol, hypertension, diabetes, and smoking status, interaction testing revealed no heterogeneity (all *P* > 0.05) (Figure 3).

### Sensitivity analysis

Considering the probable effect of using cholesterol-lowering medication on the association between RC and the risks of HF, we performed a sensitivity analysis to determine this association after excluding the participants who took the cholesterol-lowering medication (*n* = 305). In the multivariable-adjusted Cox model, the HR (CIs) for incident HF comparing the RC level of 0.50 to 0.99 mmol/L, 1.00 to 1.49 mmol/L, and ≥1.50 mmol/L with the RC < 0.50 mmol/L were 1.17 (95% CI: 1.05–1.30), 1.27(95% CI: 1.08–1.49), and 1.50 (95% CI: 1.14–1.97), respectively (*P* for trend < 0.001) (Supplementary Table 2). Furthermore, consistent with the analysis using the cubic spline curves, the risk of HF increased in participants with higher RC levels (Supplementary Figure 3). Therefore, the robustness of the association between RC and the risks of HF was supported by the consistency of results between the sensitivity analysis and the primary analysis.

## Discussion

In the present study, by conducting a secondary analysis of the ARIC study, we found that a higher level of RC was significantly associated with a higher risk of HF in the general population without coronary heart disease independent of traditional cardiovascular risk factors after a median follow-up of 22.5 years. This association was robust in the analysis of cubic spline curve and sensitivity analysis, indicating that a higher RC level might be a potential risk factor for HF events. Therefore, the management of blood cholesterol to reduce the risks of HF should focus not only on the traditional blood lipid parameters (LDL-c, non-HDL-c, or HDL-c) but also on the RC level.

Previous studies have demonstrated the detrimental effect of elevated RC on cardiovascular outcomes. Castañer et al. analyzed the association between the baseline lipid profile and major adverse cardiovascular events in the high-risk primary prevention PREDIMED (Prevención con Dieta Mediterránea) trial population. They found that the level of estimated RC, but not LDL-c or HDL-c, were independently associated with cardiovascular outcomes and suggested that RC should be considered a preferential treatment target (17). Similarly, Langsted and his colleagues included 2,973 individuals with myocardial infarction or ischemic stroke from the Copenhagen General Population Study and found that a lower RC level was estimated to reduce recurrent major adverse cardiovascular events in the secondary prevention and indicated an unmet medical need for secondary prevention in patients with high RC levels (18). In addition, Wadström and his colleagues studied 106,937 individuals from the Copenhagen General Population Study and 13,974 individuals from the Copenhagen City Heart Study and found that elevated RC was associated with an approximately 5-fold increased risk of peripheral artery disease, 3-fold increased risk of ischemic heart disease and 2-fold increased risk of ischemic stroke (9). Other investigations also demonstrated the relationship between elevated RC and a higher risk of aortic valve stenosis, cardiac allograft vasculopathy, non-alcoholic fatty liver disease, diabetic nephropathy and chronic kidney disease (10, 19–22). To the best of our knowledge, we are the first to report the association between elevated RC and the higher risk of HF in the general population. It adds to evidence that the higher RC level might be a potential risk factor for HF. It might be informative, therefore, to restrain the RC level for optimal blood cholesterol management in the prevention of long-term HF risks.

The increased risk of HF associated with the higher level of RC might be attributed to biological pathophysiological mechanisms, including atherosclerotic plaque formation and inflammation (6). As mentioned above, RC was the cholesterol content of the triglyceride-rich lipoproteins composed of very-low-density lipoproteins and intermediate-density lipoproteins in the fasting state and of these two lipoproteins together with chylomicron remnants in the non-fasting state (7). It was reported that RC was able to enter the arterial intima and might easier get trapped in the intima and taken up by macrophages compared to LDL due to the relatively larger size, making it more difficult for RC to diffuse back into the vessels because of the steep blood pressure gradient from the arterial lumen to the adventitia (23, 24). In addition, the higher level of RC might be capable of stimulating an inflammatory response and induce pro-inflammatory cytokines including tumor necrosis factor-α (TNF-α) and interleukins, which further contribute to the progression of intravascular plaque rupture thereafter HF event (25). Inflammation also contributes to the pathogenesis and progression of HF through diverse mechanistic pathways, including proinflammatory cytokines, components of the innate and humoral immune response, etc (26). The proinflammatory cytokines interleukin-1 and TNF-α both induce systolic and diastolic dysfunction; the latter also promotes adverse cardiac remodeling (27, 28). Therefore, the higher level of RC might also have a direct impact on the cardiac structure and function. Recently, several therapeutic strategies aiming to lower triglycerides and RC have been promoted, which include high-intensity statin, fibrates, PCSK9 inhibitors, et al. (29–31). However, whether these therapeutic approaches indeed bring clinical benefits to patients, namely, an independent reduction in the incidence of HF, or the improvement in long-term survival, needs further investigations in prospective and randomized analyses. Taken together, these data suggest that RC might be considered both a pragmatic prognostic predictor of HF and a potential therapeutic target for future intervention.

We should note that our study had several limitations. First, although multivariable-adjusted regression analyses were conducted, and our results remained robust in the sensitivity analyses, the observational nature of our study may potentially lead to residual confounding and therefore does not enable conclusions about the causal relationship between RC and the risks of HF. Second, the calculation of the RC was not as precise as directly measured RC, and we could not acquire the specific components of RC, such as very-low-density lipoproteins. However, the calculation of the RC from the baseline lipid profiles is easy to perform and could be conveniently applied in routine clinical practice at no extra expense. Third, although the participants with coronary heart disease were excluded in this study, we cannot evaluate the impacts of ischemic heart disease on the association between RC and the risks of HF. Thus, further research is warranted to determine if ischemic heart disease mediates this association. Finally, our study was based only on the general population that came from the ARIC study; thus, our findings need further external validation before being generalized to other populations. Our findings should be considered explorative, but it provides a depiction of the relationship between a higher level of RC and the long-term risk of HF in the general population. In addition, our findings could provide mechanistic insights into how the RC might affect the prognosis of patients, which helps in clinical decision making and could be considered as a springboard for future investigations focused on the RC.

## Conclusion

In conclusion, our study found that elevated RC was associated with a higher risk of HF in the general population. The RC level could be considered as a pragmatic predictor of HF and a potential therapeutic target. Therefore, the management of blood cholesterol to reduce the risks of HF should focus not only on the traditional blood lipid parameters (LDL-c, non-HDL-c, or HDL-c) but also on the RC level.

## Data availability statement

Publicly available datasets were analyzed in this study. This data can be found here: The ARIC study coordinating center (https://sites.cscc.unc.edu/aric/).

## Ethics statement

The studies involving human participants were reviewed and approved by the Institutional Review Boards at all participating Institutions of ARIC study (Forsyth County, North Carolina; Jackson, Mississippi; Washington County, Maryland; and Northwestern suburbs of Minneapolis, Minnesota). The patients/participants provided their written informed consent to participate in this study.

## Author contributions

HL and JHL had full access to all of the data in the study, took responsibility for the integrity of the data and the accuracy of the data analysis, and contributed to concept and design. HL, JZ, ZL, JEL, and SL contributed to acquisition, analysis, and interpretation of data. HL contributed to drafting of the manuscript. JZ, ZL, and JEL contributed to statistical analysis. JHL contributed to administrative, technical, and material support and supervision. All authors contributed to the critical revision of the manuscript for important intellectual content.

## References

[1] Yancy CW Jessup M Bozkurt B Butler J Casey DJ Colvin MM 2017 ACC/AHA/HFSA focused update of the 2013 ACCF/AHA Guideline for the management of heart failure: a report of the american college of cardiology/american heart association task force on clinical practice guidelines and the heart failure society of america. *Circulation.* (2017) 136:e137–61. 10.1161/CIR.0000000000000509 28455343

[2] McDonagh TA Metra M Adamo M Gardner RS Baumbach A Böhm M 2021 ESC Guidelines for the diagnosis and treatment of acute and chronic heart failure. *Eur Heart J.* (2021) 42:3599–726. 34447992 10.1093/eurheartj/ehab368

[3] Velagaleti RS Massaro J Vasan RS Robins SJ Kannel WB Levy D. Relations of lipid concentrations to heart failure incidence: the Framingham Heart Study. *Circulation.* (2009) 120:2345–51. 10.1161/CIRCULATIONAHA.109.830984 19933936 PMC3600834

[4] Varbo A Nordestgaard BG. Nonfasting triglycerides, low-density lipoprotein cholesterol, and heart failure risk: Two cohort studies of 113 554 individuals. *Arterioscler Thromb Vasc Biol.* (2018) 38:464–72. 10.1161/ATVBAHA.117.310269 29097364

[5] Preiss D Campbell RT Murray HM Ford I Packard CJ Sattar N The effect of statin therapy on heart failure events: a collaborative meta-analysis of unpublished data from major randomized trials. *Eur Heart J.* (2015) 36:1536–46. 10.1093/eurheartj/ehv072 25802390 PMC4769322

[6] Sandesara PB Virani SS Fazio S Shapiro MD. The forgotten lipids: triglycerides, remnant cholesterol, and atherosclerotic cardiovascular disease risk. *Endocr Rev.* (2019) 40:537–57. 10.1210/er.2018-00184 30312399 PMC6416708

[7] Twickler TB Dallinga-Thie GM Cohn JS Chapman MJ. Elevated remnant-like particle cholesterol concentration: a characteristic feature of the atherogenic lipoprotein phenotype. *Circulation.* (2004) 109:1918–25. 10.1161/01.CIR.0000125278.58527.F3 15117861

[8] Kexin W Yaodong D Wen G Rui W Jiaxin Y Xiaoli L Association of increased remnant cholesterol and the risk of coronary artery disease: A retrospective study. *Front Cardiovasc Med.* (2021) 8:740596. 10.3389/fcvm.2021.740596 34778402 PMC8585757

[9] Wadström BN Wulff AB Pedersen KM Jensen GB Nordestgaard BG. Elevated remnant cholesterol increases the risk of peripheral artery disease, myocardial infarction, and ischaemic stroke: a cohort-based study. *Eur Heart J.* (2021) 43:3258–69. 10.1093/eurheartj/ehab705 34661640

[10] Kaltoft M Langsted A Nordestgaard BG. Triglycerides and remnant cholesterol associated with risk of aortic valve stenosis: Mendelian randomization in the copenhagen general population study. *Eur Heart J.* (2020) 41:2288–99. 10.1093/eurheartj/ehaa172 32267934

[11] Xie G Zhong Y Yang S Zou Y. Remnant cholesterol is an independent predictor of new-onset diabetes: A single-center cohort study. *Diabetes Metab Syndr Obes.* (2021) 14:4735–45. 10.2147/DMSO.S341285 34887671 PMC8652915

[12] The ARIC Investigators. The atherosclerosis risk in communities (ARIC) study: design and objectives. *Am J Epidemiol.* (1989) 129:687–702. 10.1093/oxfordjournals.aje.a115184 2646917

[13] Varbo A Benn M Tybjærg-Hansen A Jørgensen AB Frikke-Schmidt R Nordestgaard BG. Remnant cholesterol as a causal risk factor for ischemic heart disease. *J Am Coll Cardiol.* (2013) 61:427–36. 10.1016/j.jacc.2012.08.1026 23265341

[14] Levey AS Stevens LA Schmid CH Zhang YL Castro AR Feldman HI A new equation to estimate glomerular filtration rate. *Ann Intern Med.* (2009) 150:604–12. 10.7326/0003-4819-150-9-200905050-00006 19414839 PMC2763564

[15] Loehr LR Rosamond WD Chang PP Folsom AR Chambless LE. Heart failure incidence and survival (from the Atherosclerosis Risk in Communities study). *Am J Cardiol.* (2008) 101:1016–22. 10.1016/j.amjcard.2007.11.061 18359324

[16] Rosamond WD Chang PP Baggett C Johnson A Bertoni AG Shahar E Classification of heart failure in the atherosclerosis risk in communities (ARIC) study: a comparison of diagnostic criteria. *Circ Heart Fail.* (2012) 5:152–9. 10.1161/CIRCHEARTFAILURE.111.963199 22271752 PMC3326579

[17] Castañer O Pintó X Subirana I Amor AJ Ros E Hernáez Á Remnant cholesterol, not LDL cholesterol, is associated with incident cardiovascular disease. *J Am Coll Cardiol.* (2020) 76:2712–24. 10.1016/j.jacc.2020.10.008 33272365

[18] Langsted A Madsen CM Nordestgaard BG. Contribution of remnant cholesterol to cardiovascular risk. *J Intern Med.* (2020) 288:116–27. 10.1111/joim.13059 32181933

[19] Alyaydin E Pogoda C Dell AA Martens S Tuleta I Reinecke H Cardiac allograft vasculopathy in a long-term follow-up after heart transplantation: Role of remnant cholesterol in residual inflammation. *Cardiol J.* (2022) 29:782–90. 10.5603/CJ.a2022.0013 35373329 PMC9550326

[20] Zou Y Lan J Zhong Y Yang S Zhang H Xie G. Association of remnant cholesterol with nonalcoholic fatty liver disease: a general population-based study. *Lipids Health Dis.* (2021) 20:139. 10.1186/s12944-021-01573-y 34657611 PMC8520640

[21] Yu D Wang Z Zhang X Qu B Cai Y Ma S Remnant cholesterol and cardiovascular mortality in patients with type 2 diabetes and incident diabetic nephropathy. *J Clin Endocrinol Metab.* (2021) 106:3546–54. 10.1210/clinem/dgab533 34291804

[22] Yan P Xu Y Miao Y Bai X Wu Y Tang Q Association of remnant cholesterol with chronic kidney disease in middle-aged and elderly Chinese: a population-based study. *Acta Diabetol.* (2021) 58:1615–25. 10.1007/s00592-021-01765-z 34181081

[23] Nordestgaard BG Tybjaerg-Hansen A Lewis B. Influx in vivo of low density, intermediate density, and very low density lipoproteins into aortic intimas of genetically hyperlipidemic rabbits. Roles of plasma concentrations, extent of aortic lesion, and lipoprotein particle size as determinants. *Arterioscler Thromb.* (1992) 12:6–18. 10.1161/01.atv.12.1.6 1731859

[24] Nordestgaard BG. The vascular endothelial barrier–selective retention of lipoproteins. *Curr Opin Lipidol.* (1996) 7:269–73. 10.1097/00041433-199610000-00002 8937515

[25] Shin HK Kim YK Kim KY Lee JH Hong KW. Remnant lipoprotein particles induce apoptosis in endothelial cells by NAD(P)H oxidase-mediated production of superoxide and cytokines via lectin-like oxidized low-density lipoprotein receptor-1 activation: prevention by cilostazol. *Circulation.* (2004) 109:1022–8. 10.1161/01.CIR.0000117403.64398.53 14967724

[26] Murphy SP Kakkar R McCarthy CP Januzzi JJ. Inflammation in Heart Failure: JACC State-of-the-Art Review. *J Am Coll Cardiol.* (2020) 75:1324–40. 10.1016/j.jacc.2020.01.014 32192660

[27] Hori M Yamaguchi O. Is tumor necrosis factor-α friend or foe for chronic heart failure? *Circ Res.* (2013) 113:492–4. 10.1161/CIRCRESAHA.113.302024 23948582

[28] Van Tassell BW Toldo S Mezzaroma E Abbate A. Targeting interleukin-1 in heart disease. *Circulation.* (2013) 128:1910–23. 10.1161/CIRCULATIONAHA.113.003199 24146121 PMC3938092

[29] Vallejo-Vaz AJ Fayyad R Boekholdt SM Hovingh GK Kastelein JJ Melamed S Triglyceride-Rich lipoprotein cholesterol and risk of cardiovascular events among patients receiving statin therapy in the TNT trial. *Circulation.* (2018) 138:770–81. 10.1161/CIRCULATIONAHA.117.032318 29618599

[30] Jun M Foote C Lv J Neal B Patel A Nicholls SJ Effects of fibrates on cardiovascular outcomes: a systematic review and meta-analysis. *Lancet.* (2010) 375:1875–84. 10.1016/S0140-6736(10)60656-3 20462635

[31] Bhatt DL Steg PG Miller M Brinton EA Jacobson TA Ketchum SB Cardiovascular risk reduction with icosapent ethyl for hypertriglyceridemia. *N Engl J Med.* (2019) 380:11–22. 10.1056/NEJMoa1812792 30415628

