# Supplementary material for: Association between remnant cholesterol and heart failure: A prospective cohort study
Source: Front Cardiovasc Med. 2022 Oct 28;9:938647. doi: 10.3389/fcvm.2022.938647 (PMC9649897; doi:10.3389/fcvm.2022.938647)
Supplement: Supplementary file 1 [file Data_Sheet_1.PDF]

Supplementary Material

1 Supplementary Figures and Tables

1.1 Supplementary Figures

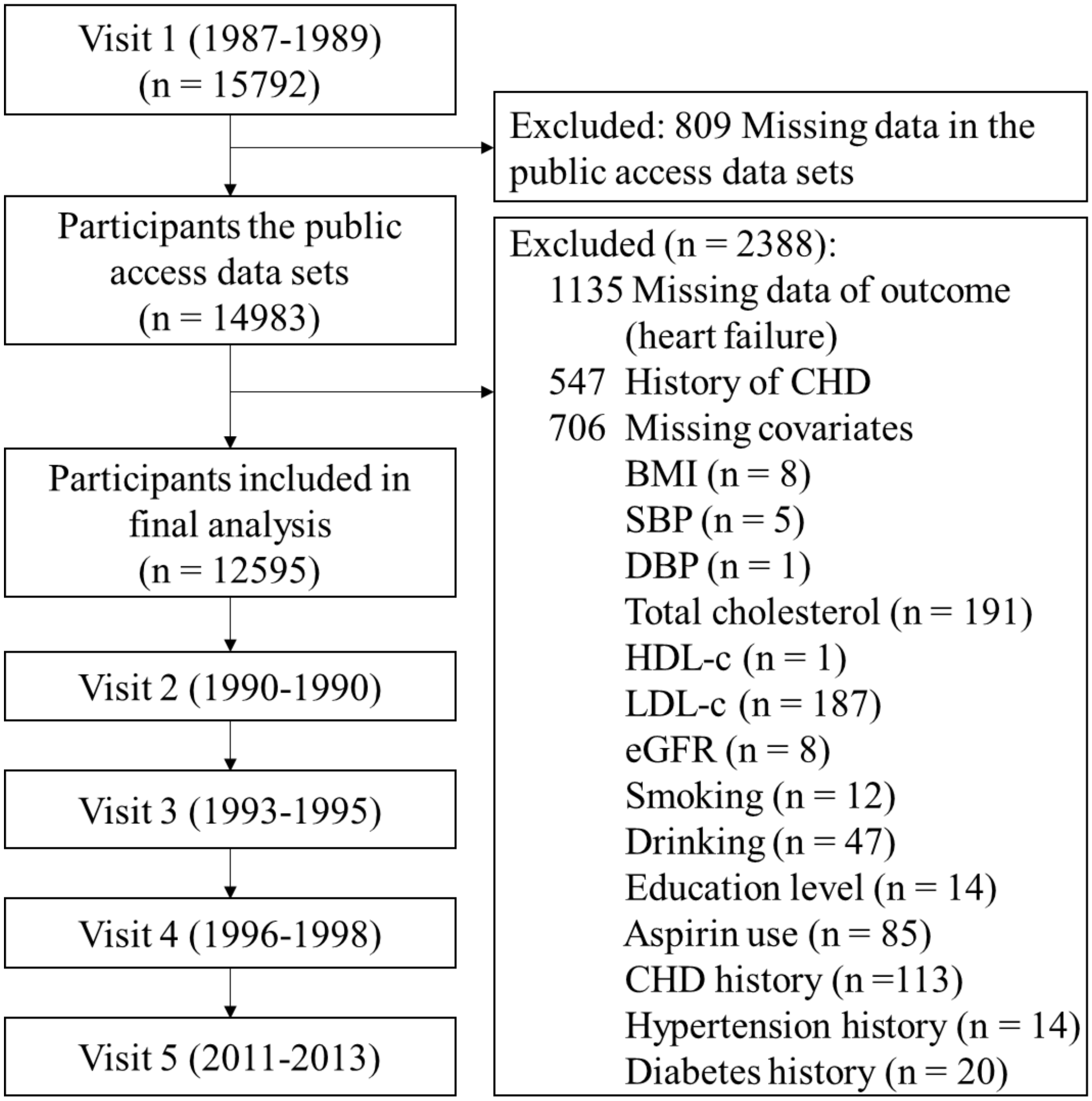

Supplementary Figure 1. Study flowchart with detailed exclusion information of participants.

CHD: coronary heart disease; BMI: body mass index; SBP: systolic blood pressure; DBP: diastolic blood pressure; HDL-c: high-density lipoprotein cholesterol; LDL-c: low-density lipoprotein cholesterol; eGFR: estimated glomerular filtration rate.

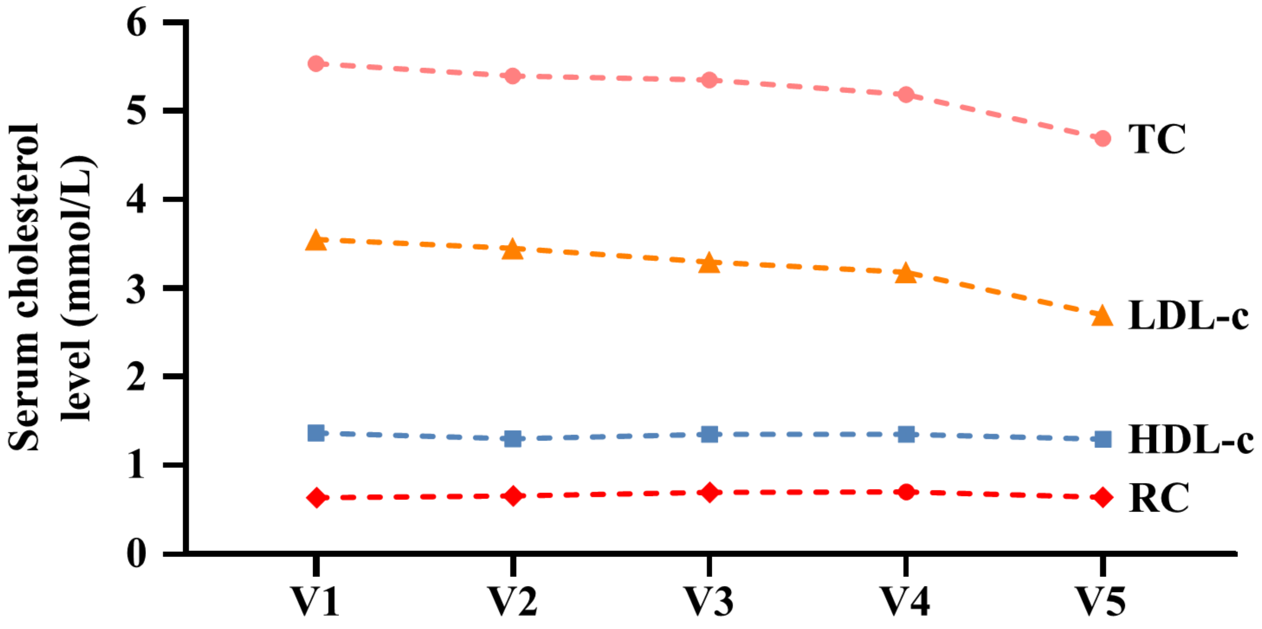

Supplementary Figure 2. The long-term changes of serum cholesterol (TC, LDL-c, HDL-c, and RC) from visit 1 to 5.

The serum cholesterol level is presented as mean. TC: total cholesterol; LDL-c: low-density lipoprotein cholesterol; HDL-c: high-density lipoprotein cholesterol; RC: remnant cholesterol.

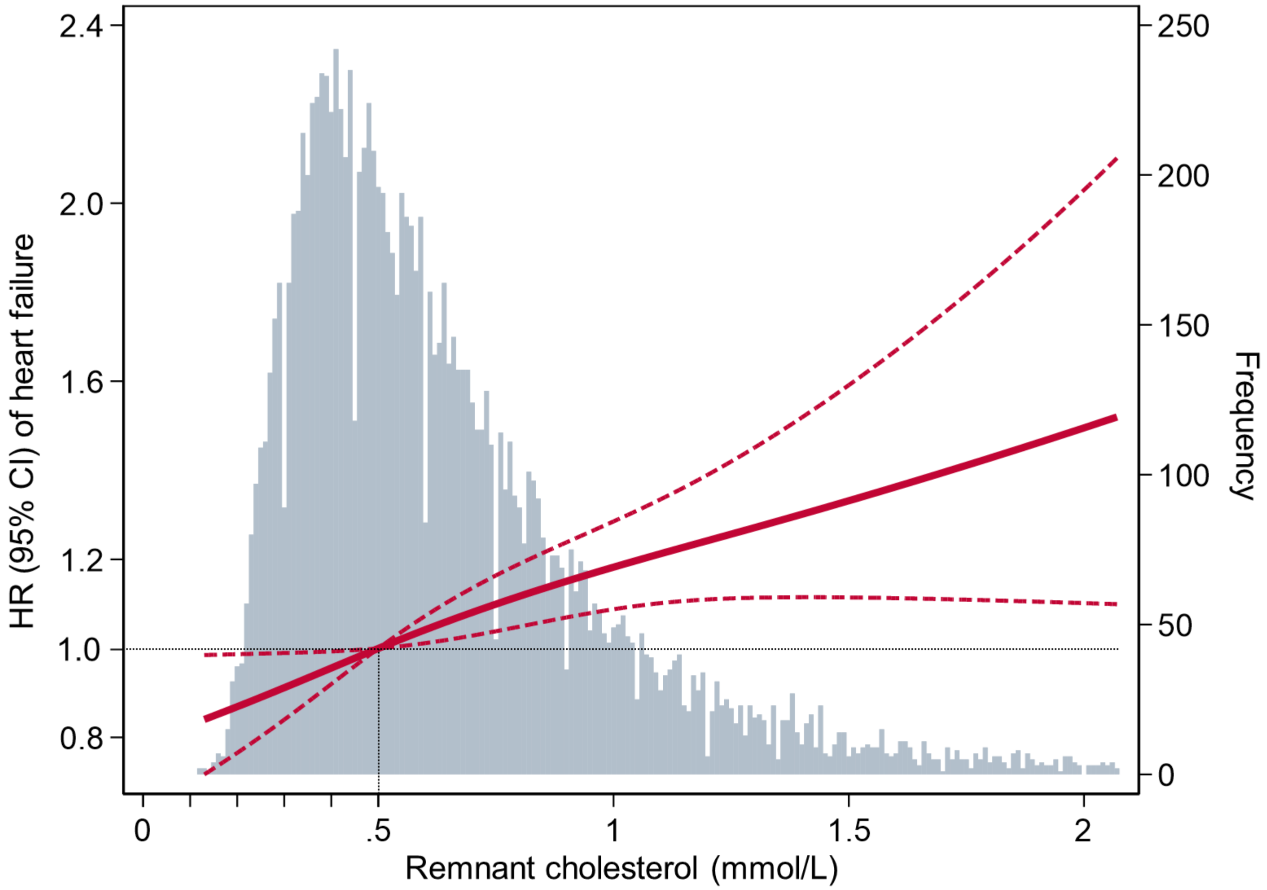

Supplementary Figure 3. Adjusted hazard ratios (95% confidence interval) for the association of the remnant cholesterol level with the risk of heart failure in participants without using cholesterol-lowering medication (n =12,290).

Hazard ratios (indicated by a dark-red solid line) and 95% confidence intervals (dark-red dotted lines) are derived from the multivariable-adjusted Cox regression model, adjusted for age, sex, race, education level, body mass index, smoking status, drinking status, systolic blood pressure, diastolic blood pressure, estimated glomerular filtration rate, prevalent hypertension, prevalent diabetes mellitus, use of aspirin and antihypertensive drugs, total cholesterol, low-density lipoprotein cholesterol. The remnant cholesterol level was centered at 0.5 mmol/L and modeled using a restricted

cubic spline with knots at the 5th, 50th, and 95th percentiles. The black dotted line is the reference line as hazard ratio = 1. Histograms represent the frequency distribution of remnant cholesterol.

1.2 Supplementary Tables

Supplementary Table 1. Association of remnant cholesterol as a continuous variable (Per 1-SD = 0.33 mmol/L) with the risk of heart failure.

Risk of heart failure	Hazard Ratio (95% CI)	*P* Value
Model 1	1.28 (1.24-1.34)	< 0.001
Model 2	1.07 (1.03-1.12)	0.001
Model 3	1.10 (1.04-1.15)	0.001

Model 1: adjusted for age, sex, race;
Model 2: adjusted for model 1 + education level, body mass index, smoking status, drinking status, systolic blood pressure, diastolic blood pressure, estimated glomerular filtration rate, prevalent hypertension, prevalent diabetes mellitus, use of aspirin and antihypertensive drugs;
Model 3: adjusted for model 2 + total cholesterol, low-density lipoprotein cholesterol, use of cholesterol-lowering medication.

Supplementary Table 2. Association of remnant cholesterol with the risk of heart failure in participants without using cholesterol-lowering medication (n =12,290).

Remnant cholesterol (mmol/L)	Model 1		Model 2		Model 3	
	Hazard Ratio (95% CI)	*P* Value	Hazard Ratio (95% CI)	*P* Value	Hazard Ratio (95% CI)	*P* Value
< 0.50	1 (ref.)	—	1 (ref.)	—	1 (ref.)	—
0.50-0.99	1.51 (1.36-1.67)	< 0.001	1.16 (1.05-1.29)	0.004	1.17 (1.05-1.30)	0.004
1.00-1.49	2.12 (1.84-2.45)	< 0.001	1.22 (1.06-1.42)	0.007	1.27 (1.08-1.49)	0.004
⩾ 1.50	2.42 (1.91-3.07)	< 0.001	1.38 (1.09-1.77)	0.009	1.50 (1.14-1.97)	0.004
P for trend	< 0.001		< 0.001		< 0.001	

Model 1: adjusted for age, sex, race;
Model 2: adjusted for model 1 + education level, body mass index, smoking status, drinking status, systolic blood pressure, diastolic blood pressure, estimated glomerular filtration rate, prevalent hypertension, prevalent diabetes mellitus, use of aspirin and antihypertensive drugs.
Model 3: adjusted for model 2 + total cholesterol, low-density lipoprotein cholesterol.
